# Supplementary material for: Fasting induces metabolic switches and spatial redistributions of lipid processing and neuronal interactions in tanycytes
Source: Nat Commun. 2024 Aug 4;15:6604. doi: 10.1038/s41467-024-50913-w (PMC11298547; doi:10.1038/s41467-024-50913-w)
Supplement: Supplementary file 1 — Supplementary Information [file 41467_2024_50913_MOESM1_ESM.pdf]

Fasting induces metabolic switches and spatial redistributions of lipid processing and neuronal interactions in tanycytes

Maxime Brunner [1,2 †], David Lopez-Rodriguez [2,3 †], Judith Estrada-Meza [2,3], Rafik Dali [2,3], Antoine Rohrbach [2,3], Tamara Deglise [2,3], Andrea Messina [1,2], Bernard Thorens [4], Federico Santoni [1,2,5 *], Fanny Langlet [2,3 *]

[1] Service of Endocrinology, Diabetology, and Metabolism, Lausanne University Hospital, 1011 Lausanne, Switzerland.
[2] Faculty of Biology and Medicine, University of Lausanne, Lausanne, Switzerland
[3] Department of Biomedical Sciences, Faculty of Biology and Medicine, University of Lausanne, Lausanne, Switzerland
[4] Center for Integrative Genomics, Faculty of Biology and Medicine, University of Lausanne, Lausanne, Switzerland
[5]Institute for Genetic and Biomedical Research (IRGB) - CNR, Monserrato, Italy

[†] These authors contributed equally.

* These authors jointly supervised this work.

Corresponding authors:

Fanny Langlet, fanny.langlet@unil.ch

Federico Santoni, federico.santoni@chuv.ch

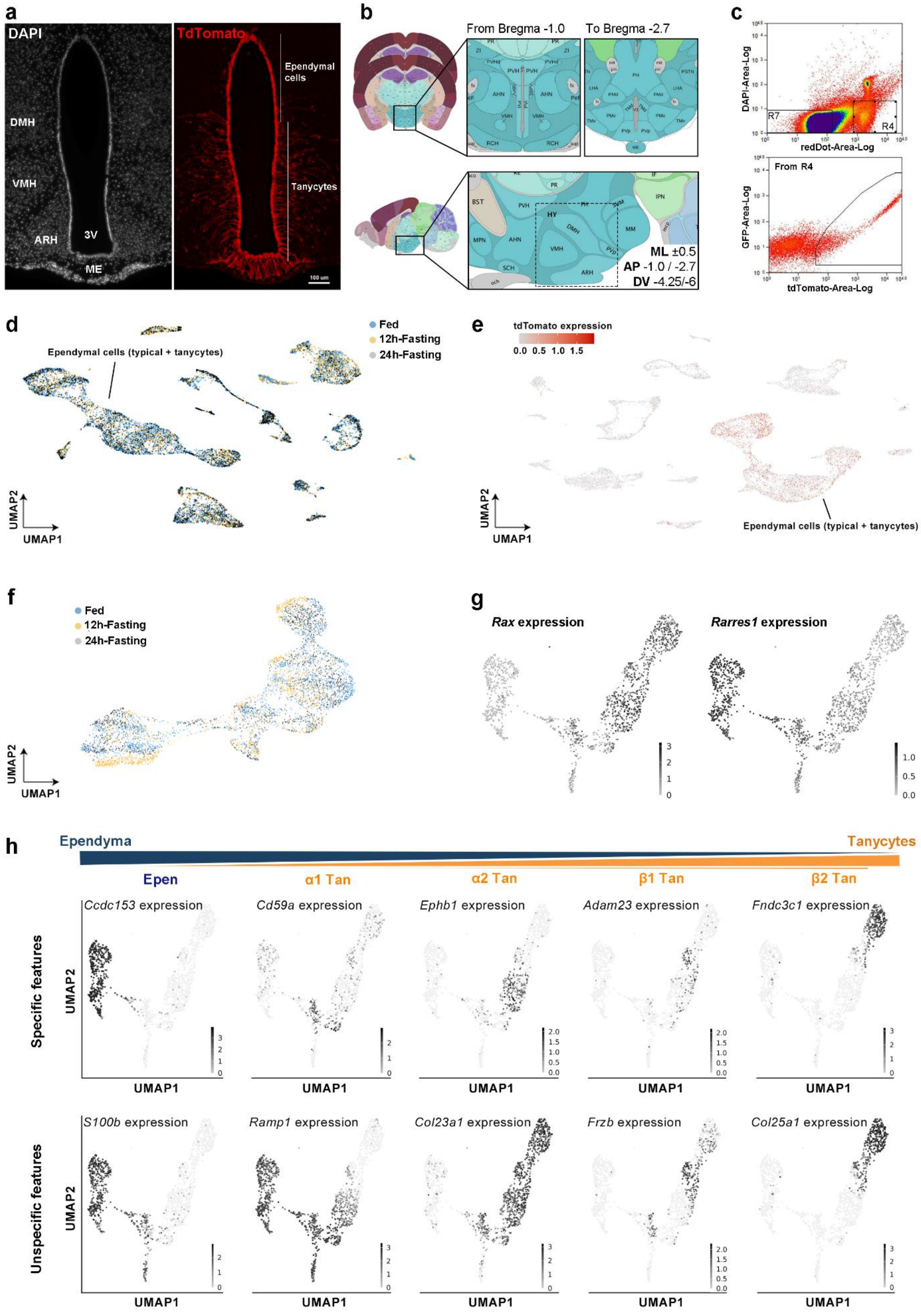
a
DAPI
TdTomato
DMH
VMH
ARH
3V
ME
Ependymal cells
Tanycytes
100 um
b
From Bregma -1.0
To Bregma -2.7
HY
ML ±0.5
AP -1.0 / -2.7
DV -4.25/-6
c
DAPI-Area-Log
redDot-Area-Log
R7
R4
From R4
GFP-Area-Log
tdTomato-Area-Log
d
Fed
12h-Fasting
24h-Fasting
Ependymal cells (typical + tanycytes)
UMAP2
UMAP1
e
tdTomato expression
0.0 0.5 1.0 1.5
Ependymal cells (typical + tanycytes)
UMAP2
UMAP1
f
Fed
12h-Fasting
24h-Fasting
UMAP2
UMAP1
g
Rax expression
Rarres1 expression
h
Ependyma
Tanycytes
Epen
α1 Tan
α2 Tan
β1 Tan
β2 Tan
Specific features
Ccdc153 expression
Cd59a expression
Ephb1 expression
Adam23 expression
Fndc3c1 expression
Unspecific features
S100b expression
Ramp1 expression
Col23a1 expression
Frzb expression
Col25a1 expression
UMAP2
UMAP1

Supplementary Figure 1. a DAPI counterstaining (left) and tdTomato expression (right) along the 3V after TAT-cre infusion. Tanycytes and ependymal cells were selectively targeted. **b** Schematic representation of the microdissected region. Images are adapted from the Mouse Brain Allen Brain Atlas. **c** Gating strategy for fluorescence-activated cell sorting (FACS). Cells were sorted according to their negativity for DAPI (viability dye), their positivity for RedDot 1 (viability dye), and their level of tdTomato fluorescence with a loose gating strategy. **d** UMAP representation of the three integrated conditions (i.e., fed, 12h-fasting, and 24h-fasting), colored by metabolic conditions. **e** tdTomato UMAP gene expression among sorted cells. **f** UMAP representation of the three integrated conditions (i.e., fed, 12h-fasting, and 24h-fasting) for the ependymal clusters, colored by metabolic conditions. **g** *Rax* and *Rarres1* UMAP gene expression in the ependymal cell populations. **h** Feature plots of specific versus unspecific genes for each ependymal cell subgroup selected from the standard clustering analysis (Supplementary Data 2).

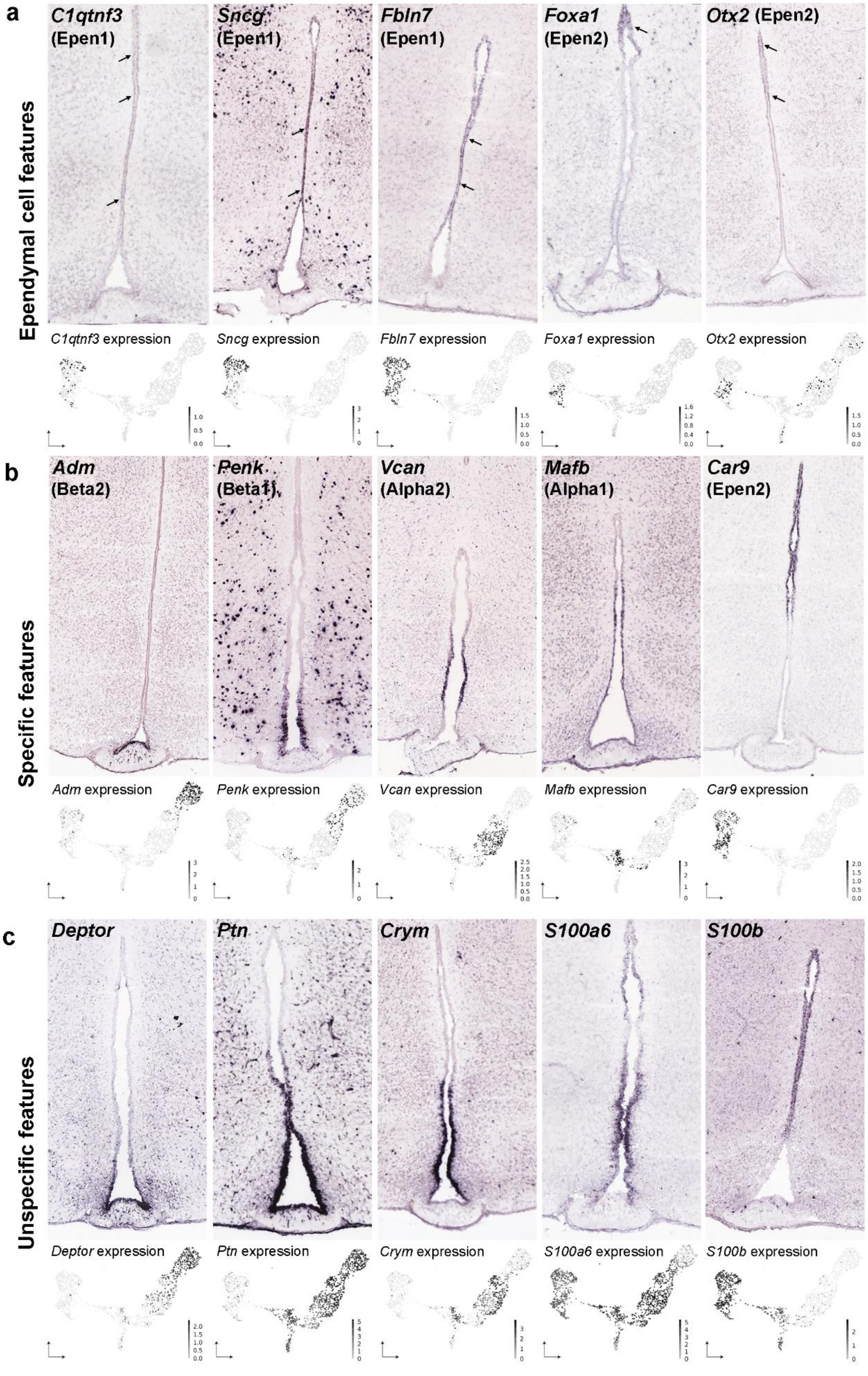

a
Ependymal cell features
C1qtnf3 (Epen1)
Sncg (Epen1)
Fbln7 (Epen1)
Foxa1 (Epen2)
Otx2 (Epen2)
C1qtnf3 expression
Sncg expression
Fbln7 expression
Foxa1 expression
Otx2 expression
b
Specific features
Adm (Beta2)
Penk (Beta1)
Vcan (Alpha2)
Mafb (Alpha1)
Car9 (Epen2)
Adm expression
Penk expression
Vcan expression
Mafb expression
Car9 expression
c
Unspecific features
Deptor
Ptn
Crym
S100a6
S100b
Deptor expression
Ptn expression
Crym expression
S100a6 expression
S100b expression

Supplementary Figure 2. Validation of specific versus unspecific features along the 3V. a-c *In situ* hybridization on coronal brain sections (obtained from Allen Mouse Brain Atlas) and feature plots derived from UMAP showing the expression and distribution of ependymal markers (a), specific markers for each ependymal subgroup (b), or unspecific features spanning different populations (c) along the 3V. The genes were selected from the standard clustering analysis (Supplementary Data 2).

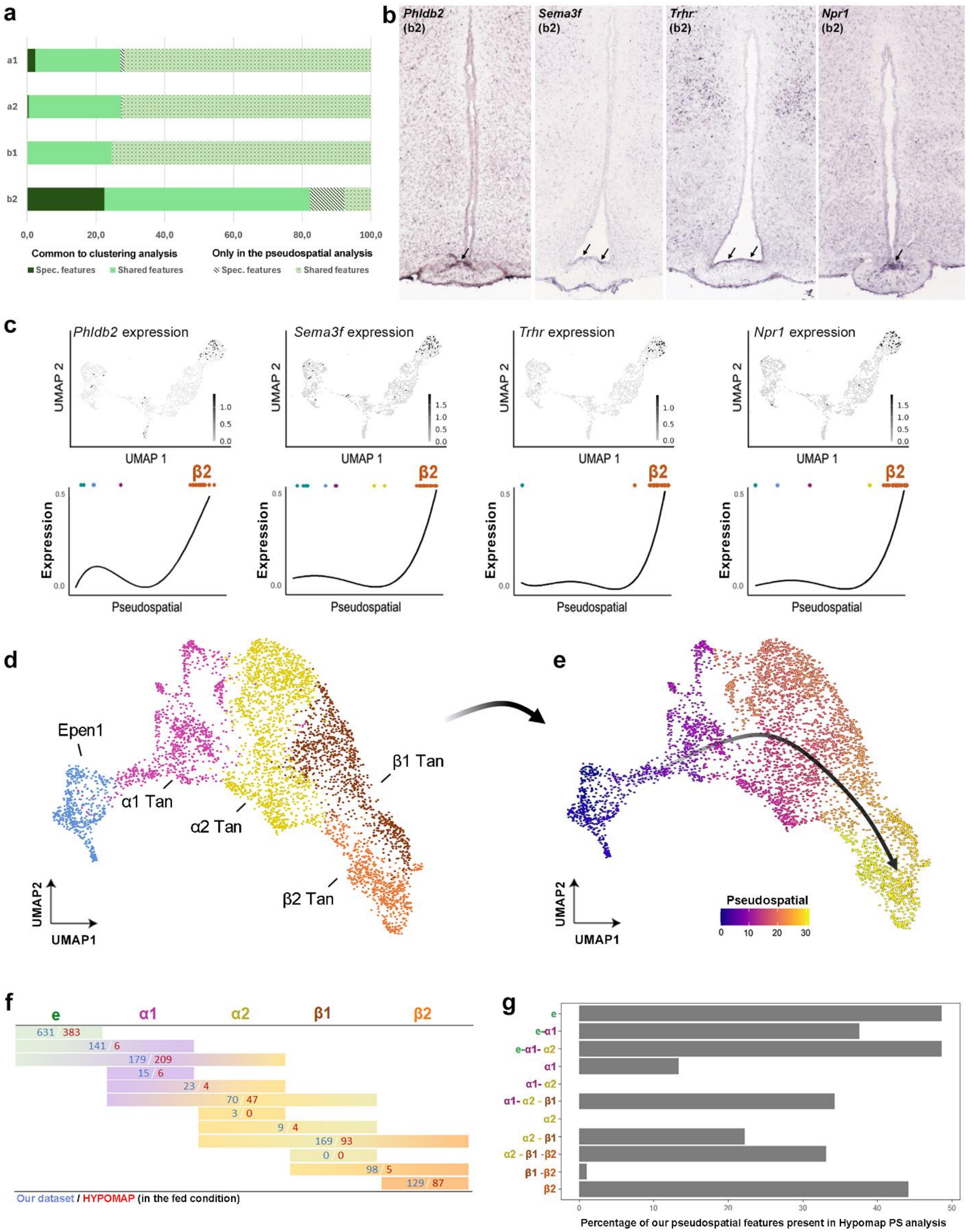
a
a1
a2
b1
b2
0,0
20,0
40,0
60,0
80,0
100,0
Common to clustering analysis
Only in the pseudospatial analysis
Spec. features
Shared features
Spec. features
Shared features
b
Phldb2 (b2)
Sema3f (b2)
Trhr (b2)
Npr1 (b2)
c
Phldb2 expression
Sema3f expression
Trhr expression
Npr1 expression
UMAP 2
UMAP 1
β2
Expression
Pseudospatial
d
Epen1
α1 Tan
α2 Tan
β1 Tan
β2 Tan
UMAP2
UMAP1
e
Pseudospatial
0
10
20
30
f
e
α1
α2
β1
β2
631 / 383
141 / 6
179 / 209
15 / 6
23 / 4
70 / 47
3 / 0
9 / 4
169 / 93
0 / 0
98 / 5
129 / 87
Our dataset / HYPOMAP (in the fed condition)
g
e
e-α1
e-α1- α2
α1
α1- α2
α1- α2 - β1
α2
α2 - β1
α2 - β1 -β2
β1 -β2
β2
Percentage of our pseudospatial features present in Hypomap PS analysis

Supplementary Figure 3. Validation for the supervised pseudospatial analysis. a Percentage of features revealed by the pseudospatial analysis compared to the standard clustering analysis. **b-c** *In situ* hybridization on coronal brain sections (b), feature plots, and pseudospatial trajectories (c) of some specific genes along the typical ependymal cells→β2-tanycytes trajectory. **d-e** UMAP representation of Hypomap cells in the fed condition (d) converting into a pseudospatial trajectory from typical ependymal cells to β2-tanycytes (e). UMAP distribution follows the anatomical ventrodorsal axis for the different ependymal subgroups (i.e., β2→β1→α2→α1→typical ependymal cells), as in our dataset. **f** Number of features significantly correlating with one (specific features) versus multiple ependymal populations (shared features) found in our dataset (blue, fed) and Hypomap dataset (red, Hypomap). **g** Percentage of features revealed by our pseudospatial analysis compared to the Hypomap dataset for each combination. See Supplementary Data 3.

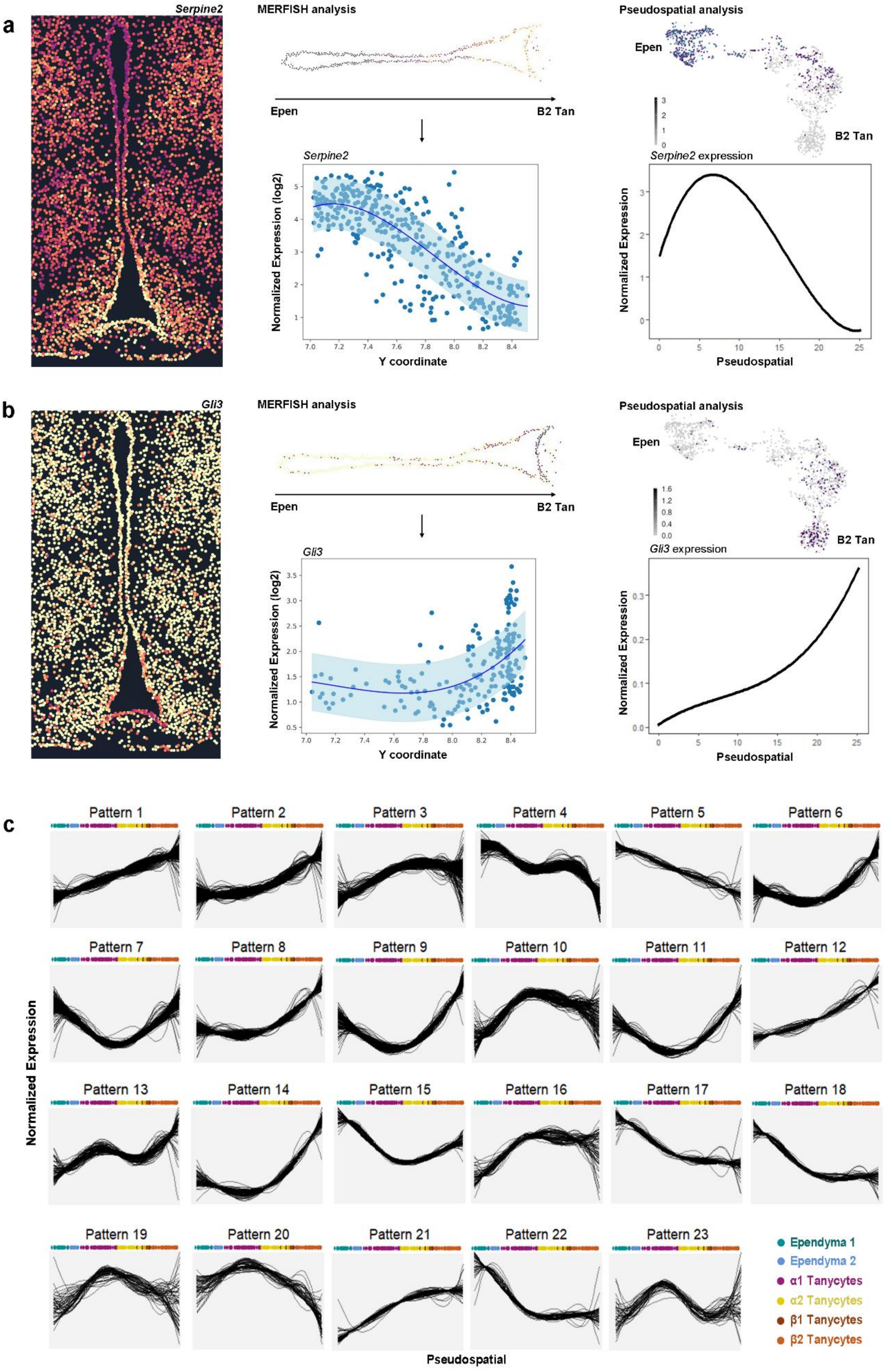

a
Serpine2
MERFISH analysis
Epen
B2 Tan
Serpine2
Normalized Expression (log2)
Y coordinate
Pseudospatial analysis
Epen
B2 Tan
Serpine2 expression
Normalized Expression
Pseudospatial
b
Gli3
MERFISH analysis
Epen
B2 Tan
Gli3
Normalized Expression (log2)
Y coordinate
Pseudospatial analysis
Epen
B2 Tan
Gli3 expression
Normalized Expression
Pseudospatial
c
Pattern 1
Pattern 2
Pattern 3
Pattern 4
Pattern 5
Pattern 6
Pattern 7
Pattern 8
Pattern 9
Pattern 10
Pattern 11
Pattern 12
Pattern 13
Pattern 14
Pattern 15
Pattern 16
Pattern 17
Pattern 18
Pattern 19
Pattern 20
Pattern 21
Pattern 22
Pattern 23
Normalized Expression
Pseudospatial
Ependyma 1
Ependyma 2
α1 Tanycytes
α2 Tanycytes
β1 Tanycytes
β2 Tanycytes

Supplementary Figure 4. a-b MERFISH (obtained from Allen brain atlas) versus pseudospatial analysis revealing the distribution of Serpine2 and Gli3 along the third ventricle. Similar trajectories were obtained with the two different approaches. **c** Twenty-three patterns of gene expression observed in the fed condition along the 3V pseudospatial trajectory obtained using TradeSeq. Only patterns with more than 50 genes are displayed. See Supplementary Data 4.

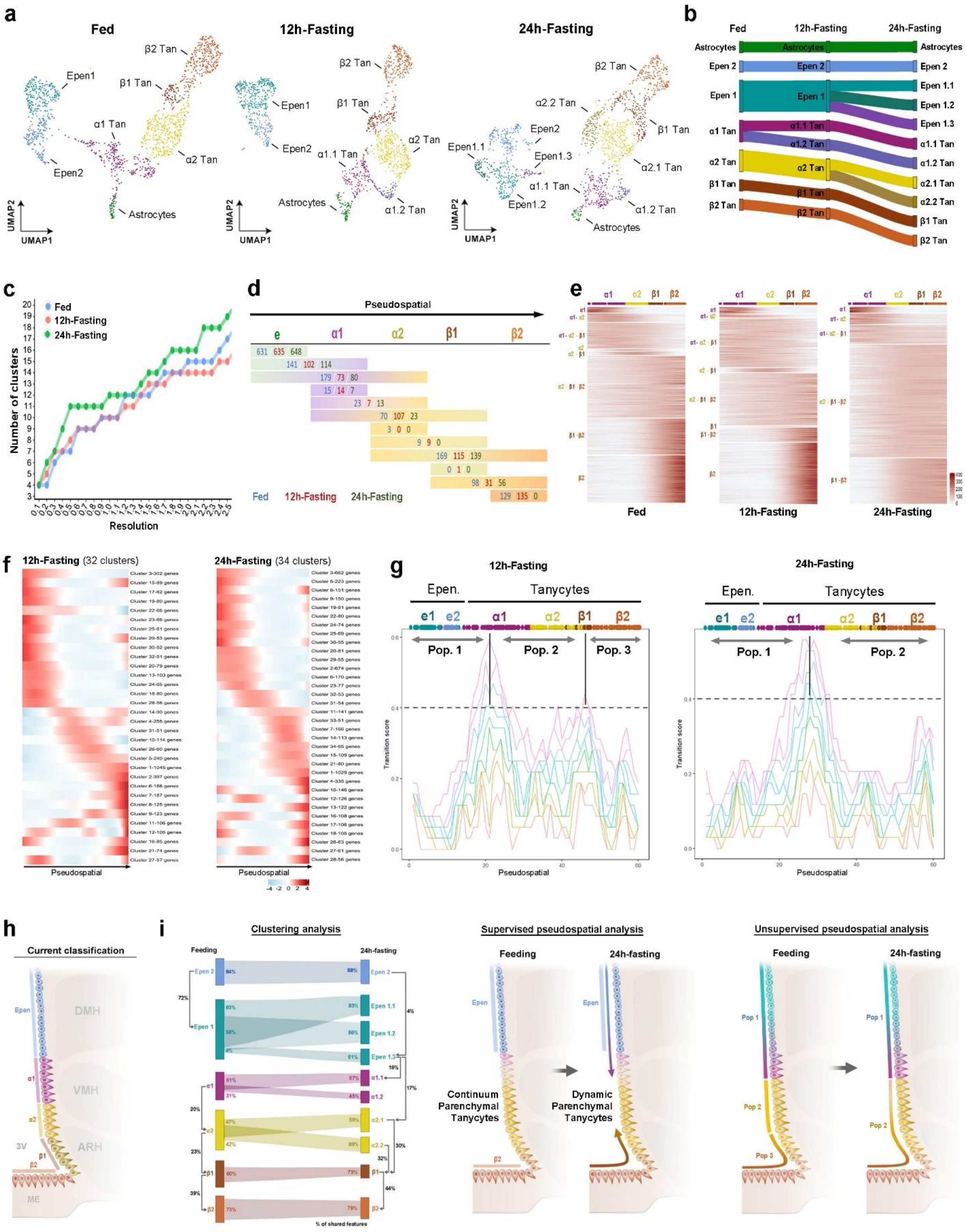

a
Fed
12h-Fasting
24h-Fasting
β2 Tan
Epen1
β1 Tan
α1 Tan
α2 Tan
Epen2
Astrocytes
α1.1 Tan
α1.2 Tan
α2.2 Tan
α2.1 Tan
Epen1.1
Epen1.2
Epen1.3
UMAP2
UMAP1
b
Fed
12h-Fasting
24h-Fasting
Astrocytes
Epen 2
Epen 1
Epen 1.1
Epen 1.2
Epen 1.3
α1 Tan
α1.1 Tan
α1.2 Tan
α2 Tan
α2.1 Tan
α2.2 Tan
β1 Tan
β2 Tan
c
Fed
12h-Fasting
24h-Fasting
Number of clusters
Resolution
d
Pseudospatial
e
α1
α2
β1
β2
631 635 648
141 102 114
179 73 80
15 14 7
23 7 13
70 107 23
3 0 0
9 9 0
169 115 139
0 1 0
98 31 56
129 135 0
Fed
12h-Fasting
24h-Fasting
e
Fed
12h-Fasting
24h-Fasting
f
12h-Fasting (32 clusters)
24h-Fasting (34 clusters)
Pseudospatial
g
12h-Fasting
24h-Fasting
Epen.
Tanycytes
e1
e2
α1
α2
β1
β2
Pop. 1
Pop. 2
Pop. 3
Transition score
Pseudospatial
h
Current classification
Epen
DMH
α1
VMH
α2
3V
ARH
β1
β2
ME
i
Clustering analysis
Feeding
24h-fasting
% of shared features
Supervised pseudospatial analysis
Feeding
24h-fasting
Epen
Continuum Parenchymal Tanycytes
Dynamic Parenchymal Tanycytes
Unsupervised pseudospatial analysis
Pop 1
Pop 2
Pop 3

Supplementary Figure 5. Increased heterogeneity along the 3V during energy imbalance.

a Clustering of tanycytes, ependymal cells, and astrocyte-like cells in the fed, 12h-fasting, and 24h-fasting conditions, colored per cluster and annotated according to known cell types. **b** Sankey diagram describing cell-type fate across metabolic conditions. **c** Number of identified clusters depending on the resolution parameter. The number of clusters increases quicker at 24h-fasting compared to the other conditions, confirming an increased heterogeneity. **d** Number of features significantly correlating with one (i.e., specific features) versus multiple (i.e., shared features) ependymal populations for each metabolic condition, determined using the supervised PSA. Fasting decreases the number of specific features. **e** Heatmaps showing the specific and shared features found in the α1→β2 tanycytes trajectory. β2 tanycytes lose their specificity. **f** Heatmaps showing different gene expression patterns along the pseudospatial trajectory in 12h-fasting and 24h-fasting, determined using the unsupervised PSA. **g** Graphs representing the main transition region along the pseudospatial trajectory. The transition score was calculated as an on-off switch in gene expression along the trajectory. The different curves represent the different on-off expression gating thresholds used for the analysis. **h-i** Representative schemas of the current ependyma classification (h) and re-classification obtained using clustering, supervised pseudospatial, and unsupervised pseudospatial analysis according to the metabolic state (i). A higher heterogeneity is observed during fasting, blunting the boundaries between the tanycyte subgroups. See Supplementary data 2,3, and 4.

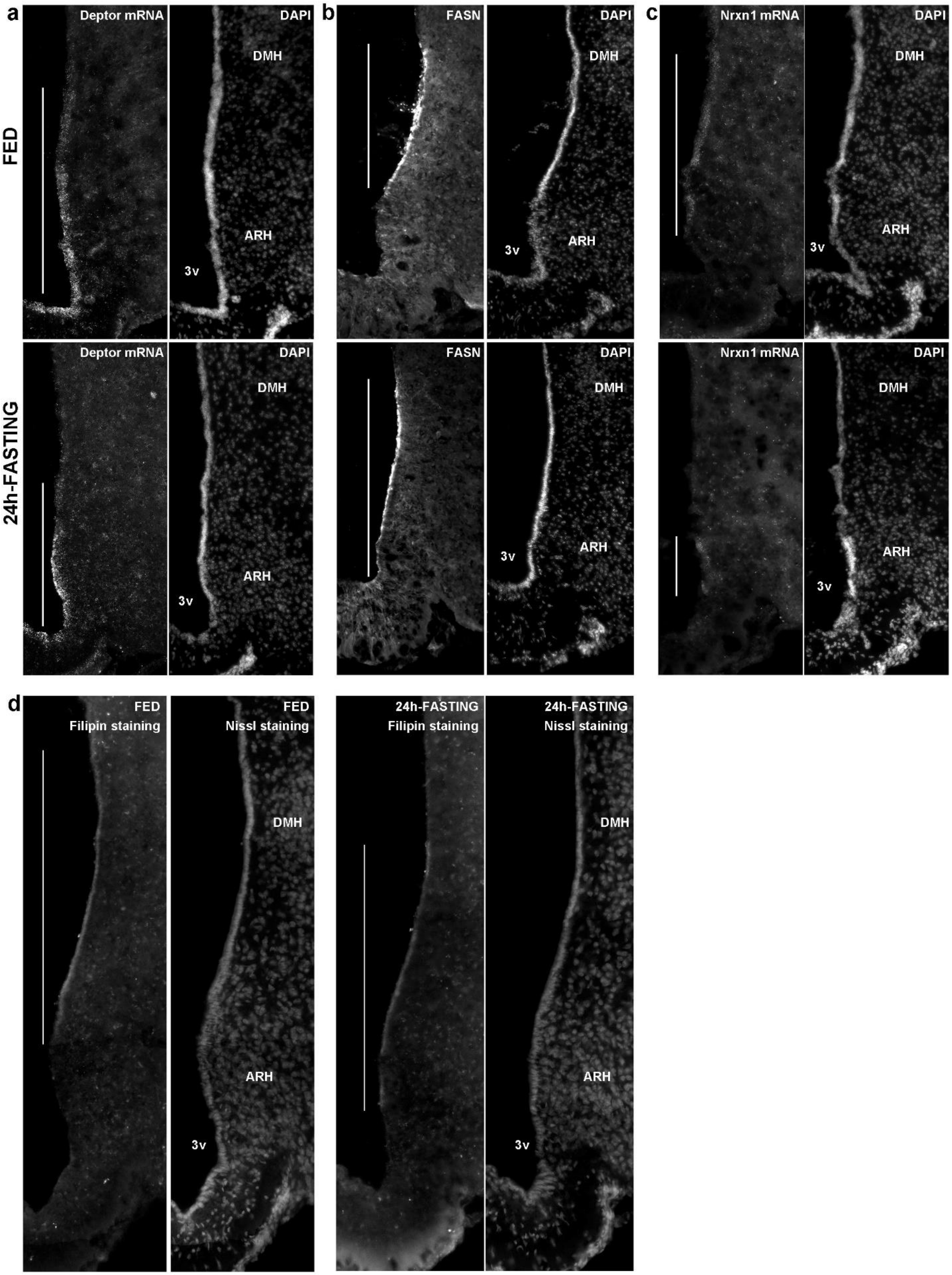
a
b
c
FED
24h-FASTING
Deptor mRNA
DAPI
FASN
Nrxn1 mRNA
DMH
ARH
3v
d
FED
Filipin staining
Nissl staining
24h-FASTING

Supplementary Figure 6. DAPI and Nissl staining for ISH and IHC images. a *Deptor* mRNA and DAPI distribution along the 3V in the fed versus 24h-fasting condition. **b** FASN protein and DAPI distribution along the 3V in the fed versus 24h-fasting condition. **c** *Nrxn1* mRNA and DAPI distribution along the 3V in the fed versus 24h-fasting condition. **d** Free cholesterol distribution using Filipin staining and Nissl staining along the 3V in the fed versus 24h-fasting condition.
