# Supplementary material for: Fasting induces metabolic switches and spatial redistributions of lipid processing and neuronal interactions in tanycytes
Source: Nat Commun. 2024 Aug 4;15:6604. doi: 10.1038/s41467-024-50913-w (PMC11298547; doi:10.1038/s41467-024-50913-w)
Supplement: Supplementary file 3 — Description of additional supplementary files [file 41467_2024_50913_MOESM3_ESM.pdf]

<u>Description of Additional Supplementary Files</u>

Supplementary Data 1. Methods. A. Experiment summary (related to Fig.1a). **B.** Cell count on the general clustering workflow (related to Fig.1b). **C.** Number of tdTomato-expressing cells on the general workflow (related to Supp. Fig. 1). **D.** Cell count on the ependyma clustering workflow (related to Fig.1d and 4a).

Supplementary Data 2. Standard clustering analysis. A-B. List of features for each cluster on the general clustering workflow (related to Fig.1c) (A) and on the ependyma clustering workflow in the fed condition (related to Fig.1e) (B). avg_logFC is the log fold-change of the average expression between the cluster of interest and all other cells (blue). Positive values indicate that the feature is more highly expressed in the first group. pct.1 and pct.2 are the percentage of cells where the feature is detected in the cluster of interest and all other cells, respectively. The pct.1/pct.2 ratio indicates the specificity (yellow). **C-G.** GO_MolecularFunction (C), GO_BiologicalProcess (D), GO_CellularComponent (E), KEGG (F), and GWAS enrichment (G) for each cluster on the ependyma clustering workflow in the fed condition (related to Fig.1f). **H.** List of features for each cluster on the ependyma clustering workflow for the fed, 12h-fasting, and 24h-fasting conditions, separately (related to Supp. Fig. 5). **I.** Number and percentage of common features between subgroups and metabolic conditions used to build the Sankey diagram (related to Supp. Fig. 5).

Supplementary Data 3. Supervised pseudospatial analysis A. List of specific (i.e., highly expressed in one population) versus shared features (i.e., spanning over multiple populations) for ependymal populations in the fed, 12h-fasting, and 24h-fasting conditions (related to Fig.2d and Supp. Fig.5). **B.** Comparison of features between the standard clustering and pseudospatial analyses (related to Supp. Fig. 3). **C-E.** GO_MolecularFunction (MF), GO_BiologicalProcess (BP), GO_CellularComponent (CC), KEGG, and GWAS enrichment for each supervised PS classification in the fed (C), 12h-fasting (D), and 24h-fasting (E) conditions (related to Fig.2d and Supp. Fig. 5). **F.** Percentage of cells co-expressing shared b2_b1 and b1_a2 features cells for each tanycyte subgroup in the fed condition. **G-H**. List of specific versus shared features (G) and GOs (H) for each ependymal population in the mouse Hypomap dataset in the fed condition (related to Supp. Fig. 3).

Supplementary Data 4. Unsupervised pseudospatial analysis. A. List of features in the ventrodorsal gene expression patterns along the third ventricle in the fed, 12h-fasting, and 24h-fasting conditions (related to Fig.3b and Supp. Fig. 5). **B.** GO_MolecularFunction (MF), GO_BiologicalProcess (BP), GO_CellularComponent (CC), KEGG, and GWAS enrichment for each pattern along the ependyma in the fed condition (related to Fig.5b).

Supplementary Data 5. Differential gene expression analysis. A. List of features for each cluster on the ependyma clustering workflow for the integrated fed, 12h-fasting, and 24h-fasting conditions (related to Fig.4a). avg_logFC is the log fold-change of the average expression between the cluster of interest and all other cells (blue). Positive values indicate that the feature is more highly expressed in the first group. pct.1 and pct.2 are the percentage of cells where the feature is detected in the cluster of interest and all other cells, respectively. The pct.1/pct.2 ratio indicates the specificity (yellow). **B-D.** List of differentially expressed genes for each cluster in fed *vs.* 12h-fasting (B), fed *vs.* 24h-fasting (C), and 12h-fasting *vs.* 24h-fasting (D) on the ependyma clustering workflow (related to Fig.4c). **E.** List of features for each temporal trajectory from fed to 24h-fasting (related to Fig.4e). **F-J.** GO_MolecularFunction (F), GO_BiologicalProcess (G), GO_CellularComponent (H), KEGG (I), and GWAS enrichment (J) for each temporal trajectory on the ependyma clustering workflow (related to Fig.4f).

Supplementary Data 6. Cell-Cell communications for Tanycytes↔Neurons. A. List of differentially regulated ligand-receptor couples in fed *vs.* 12h-fasting, fed *vs.* 24h-fasting, and 12h *vs.* 24h-fasting conditions between tanycytes and neurons (related to Fig.7b).
