# Supplementary material for: Fasting induces metabolic switches and spatial redistributions of lipid processing and neuronal interactions in tanycytes
Source: Nat Commun. 2024 Aug 4;15:6604. doi: 10.1038/s41467-024-50913-w (PMC11298547; doi:10.1038/s41467-024-50913-w)
Supplement: Supplementary file 10 — Reporting Summary [file 41467_2024_50913_MOESM10_ESM.pdf]

nature portfolio

Corresponding author(s): Fanny Langlet, Federico Santoni

Last updated by author(s): Jun 27, 2024

Reporting Summary

Nature Portfolio wishes to improve the reproducibility of the work that we publish. This form provides structure for consistency and transparency in reporting. For further information on Nature Portfolio policies, see our Editorial Policies and the Editorial Policy Checklist.

Statistics

For all statistical analyses, confirm that the following items are present in the figure legend, table legend, main text, or Methods section.

n/a	Confirmed	
☐	☒	The exact sample size (*n*) for each experimental group/condition, given as a discrete number and unit of measurement
☐	☒	A statement on whether measurements were taken from distinct samples or whether the same sample was measured repeatedly
☐	☒	The statistical test(s) used AND whether they are one- or two-sided<br>*Only common tests should be described solely by name; describe more complex techniques in the Methods section.*
☒	☐	A description of all covariates tested
☒	☐	A description of any assumptions or corrections, such as tests of normality and adjustment for multiple comparisons
☐	☒	A full description of the statistical parameters including central tendency (e.g. means) or other basic estimates (e.g. regression coefficient) AND variation (e.g. standard deviation) or associated estimates of uncertainty (e.g. confidence intervals)
☐	☒	For null hypothesis testing, the test statistic (e.g. *F*, *t*, *r*) with confidence intervals, effect sizes, degrees of freedom and *P* value noted<br>*Give P values as exact values whenever suitable.*
☒	☐	For Bayesian analysis, information on the choice of priors and Markov chain Monte Carlo settings
☒	☐	For hierarchical and complex designs, identification of the appropriate level for tests and full reporting of outcomes
☒	☐	Estimates of effect sizes (e.g. Cohen's *d*, Pearson's *r*), indicating how they were calculated

Our web collection on statistics for biologists contains articles on many of the points above.

Software and code

Policy information about availability of computer code

Data collection	Novel R codes are available through GitHub (https://github.com/dalodriguez/DPGEA & https://github.com/dalodriguez/ExPCom). Additional analysis were done using commonly used R packages for single cell RNAseq analysis, as Seurat, Monocle3, and TradeSeq. A processed dataset is available in these repositories ensuring the reproducibility of the results. Raw data is available in https://www.ncbi.nlm.nih.gov/geo/query/acc.cgi?acc=GSE266664.<br>The data supporting the findings are also available from the corresponding authors if needed.
Data analysis	The workflow used for the analysis in this manuscript have been extensively described in the methods section. Please refer to our github repository https://github.com/dalodriguez for details on the workflow used in the cell-cell communication analysis and the pseudospatial gradient expression analysis. Data in the github repository and the vignette allows to replicate the analysis. Additional analysis were done using commonly used R packages for single cell RNAseq analysis, as Seurat, Monocle3 and TradeSeq.

For manuscripts utilizing custom algorithms or software that are central to the research but not yet described in published literature, software must be made available to editors and reviewers. We strongly encourage code deposition in a community repository (e.g. GitHub). See the Nature Portfolio guidelines for submitting code & software for further information.

Data

Policy information about availability of data

All manuscripts must include a data availability statement. This statement should provide the following information, where applicable:

- Accession codes, unique identifiers, or web links for publicly available datasets
- A description of any restrictions on data availability
- For clinical datasets or third party data, please ensure that the statement adheres to our policy

The accession number for the original single-cell transcriptome reported in this paper is GSE266664 (Gene Expression Omnibus-GEO; https://www.ncbi.nlm.nih.gov/geo/query/acc.cgi?acc=GSE266664). The Hypomap dataset was also used, and it is publicly available at https://www.repository.cam.ac.uk/items/8f9c3683-29fd-44f3-aad5-7acf5e963a75. Source data for neuroanatomical are provided with this paper. The data supporting the findings of this study are also available from the corresponding authors upon request.

Research involving human participants, their data, or biological material

Policy information about studies with human participants or human data. See also policy information about sex, gender (identity/presentation), and sexual orientation and race, ethnicity and racism.

Reporting on sex and gender	NA
Reporting on race, ethnicity, or other socially relevant groupings	NA
Population characteristics	NA
Recruitment	NA
Ethics oversight	NA

Note that full information on the approval of the study protocol must also be provided in the manuscript.

Field-specific reporting

Please select the one below that is the best fit for your research. If you are not sure, read the appropriate sections before making your selection.

☒ Life sciences ☐ Behavioural & social sciences ☐ Ecological, evolutionary & environmental sciences

For a reference copy of the document with all sections, see nature.com/documents/nr-reporting-summary-flat.pdf

Life sciences study design

All studies must disclose on these points even when the disclosure is negative.

Sample size	The number of mice were chosen according the number of cells needed for the single cell RNAseq. For ISH/IHC/staining validation, the number of mice were chosen according past studies in our lab and in the literature.
Data exclusions	One mice was excluded for single cell RNAseq due to a technical issue during FACS (it is detailed in Supplementary Data1). For ISH/IHC validations, no mice were excluded.
Replication	Novel bioinformatic analyses (notably the pseudospatial analysis) were validated by applying it on other dataset, notably the mouse HypoMap and MERFISH data. To replicate the neuroanatomical analysis, the experiments were perform on 2 cohorts.
Randomization	It was a random distribution.
Blinding	The investigators were not blinded. Blinding was not possible as the groups were fed versus fasting mice.

Reporting for specific materials, systems and methods

We require information from authors about some types of materials, experimental systems and methods used in many studies. Here, indicate whether each material, system or method listed is relevant to your study. If you are not sure if a list item applies to your research, read the appropriate section before selecting a response.

Materials & experimental systems

n/a	Involved in the study
☐	☒ Antibodies
☒	☐ Eukaryotic cell lines
☒	☐ Palaeontology and archaeology
☐	☒ Animals and other organisms
☒	☐ Clinical data
☒	☐ Dual use research of concern
☒	☐ Plants

Methods

n/a	Involved in the study
☒	☐ ChIP-seq
☐	☒ Flow cytometry
☒	☐ MRI-based neuroimaging

Antibodies

Antibodies used

The primary antibodies used in this study are anti-FASN (Abcam, ab22759; 1:500), anti-HuC/HuD (Invitrogen, A21271; 1:200), anti-VGLUT2 (Synaptic system, 135403, 1:500), and anti-VGAT (Synaptic system, 131004, 1:500).

Validation

Knockout validated (for instance, https://www.abcam.com/products/primary-antibodies/fatty-acid-synthase-antibody-ab22759.html) and highly used antibodies in the field.

Animals and other research organisms

Policy information about studies involving animals; ARRIVE guidelines recommended for reporting animal research, and Sex and Gender in Research

Laboratory animals

2-to-4-month-old male Rosa26-floxed stop tdTomato mice (initially obtained from Jax) and C57Bl6/J mice (initially obtained from Charles River) were used in this study. Details are given in supplementary data 1. Animals were housed in groups (from 2 to 5 mice per cage) and maintained in a temperature-controlled room (at 22–23°C) on a 12 h light/dark cycle with ad libitum access to a chow diet and water.

Wild animals

NA

Reporting on sex

Data on males (indicated in the abstract, the main text, and the methods).
Study designed on males only to limit the group numbers for the technical difficulties (establishement of FACS-associated scRNAseq).

Field-collected samples

NA

Ethics oversight

All animal procedures were approved by the Veterinary Office of Canton de Vaud.

Note that full information on the approval of the study protocol must also be provided in the manuscript.

Plants

Seed stocks

NA

Novel plant genotypes

NA

Authentication

NA

Flow Cytometry

Plots

Confirm that:

☒ The axis labels state the marker and fluorochrome used (e.g. CD4-FITC).

☒ The axis scales are clearly visible. Include numbers along axes only for bottom left plot of group (a 'group' is an analysis of identical markers).

☒ All plots are contour plots with outliers or pseudocolor plots.

☒ A numerical value for number of cells or percentage (with statistics) is provided.

Methodology

Sample preparation	Mice were killed between 8 a.m. and 9 a.m. Adult male MBH were microdissected using a binocular microscope and put in 500µl ice-cold papain solution (20u/ml). Cells were then dissociated following Worthington's instructions. Briefly, cells were dissociated by incubating papain solution containing microdissected tissue at 37°C for 30 minutes, followed by gentle manual trituration. Cell suspensions were centrifuged at 330g for 5 minutes, and the cell pellet was then resuspended in a 500 µl albumin/ovomucoid protease inhibitor solution (1 mg/ml). A discontinuous density gradient centrifugation was performed by layering the cell suspension on an 800 µl albumin/ovomucoid protease inhibitor solution (10 mg/ml) and centrifuged at 100g for 7 minutes. Cell pellets were finally resuspended in 400 µl ice-cold calcium-free and magnesium-free HBSS.
Instrument	TdTomato-positive singlet cells were sorted using a Beckman Coulter Moflo Astrios FAC-sorter.
Software	Beckman Coulter Moflo Astrios FAC-sorter software
Cell population abundance	% of sorted cells on the total => 0.46% (between 0.28 and 0.66%)<br>% efficiency => 88% (between 83 and 93%)
Gating strategy	TdTomato-positive singlet cells were sorted uaccording to their Forward and Side scattering properties (FSC and SSC), their negativity for DAPI (Viability dye, Blue DNA intercalating agent, ThermoFisher, cat nb D1306, λEx/λEm (with DNA) = 358/461 nm), their positivity for RedDot1 (Viability dye, Far-red DNA intercalating agent, Biotium, #40060-1, λEx/λEm (with DNA) = 662/694 nm), and their level of tdTomato fluorescence emission (λEx/λEm = 554/581 nm). Gating parameters were set to improve the purity (i.e., fast collection, Noozle 70, purity) while keeping a loose FACS gate.

☒ Tick this box to confirm that a figure exemplifying the gating strategy is provided in the Supplementary Information.
